# Seroepidemiology of helminths and the association with severe malaria among infants and young children in Tanzania

**DOI:** 10.1371/journal.pntd.0006345

**Published:** 2018-03-26

**Authors:** Jennifer L. Kwan, Amy E. Seitz, Michal Fried, Kun-Lin Lee, Simon Metenou, Robert Morrison, Edward Kabyemela, Thomas B. Nutman, D. Rebecca Prevots, Patrick E. Duffy

**Affiliations:** 1 Epidemiology Unit, Laboratory of Clinical Infectious Disease, Division of Intramural Research (DIR), National Institute of Allergy and Infectious Diseases (NIAID), National Institutes of Health (NIH), Bethesda, MD, United States of America; 2 Laboratory of Malaria Immunology and Vaccinology, DIR, NIAID, NIH, Bethesda, MD, United States of America; 3 Laboratory of Parasitic Diseases, DIR, NIAID, NIH, Bethesda, MD, United States of America; 4 Seattle Biomedical Research Institute, Seattle, WA, United States of America; University of Sao Paolo, BRAZIL

## Abstract

The disease burden of *Wuchereria bancrofti* and *Plasmodium falciparum* malaria is high, particularly in Africa, and co-infection is common. However, the effects of filarial infection on the risk of severe malaria are unknown. We used the remaining serum samples from a large cohort study in Muheza, Tanzania to describe vector-borne filarial sero-reactivity among young children and to identify associations between exposure to filarial parasites and subsequent severe malaria infections. We identified positive filarial antibody responses (as well as positive antibody responses to *Strongyloides stercoralis*) among infants as young as six months. In addition, we found a significant association between filarial seropositivity at six months of age and subsequent severe malaria. Specifically, infants who developed severe malaria by one year of age were 3.9 times more likely (OR = 3.9, 95% CI: 1.2, 13.0) to have been seropositive for filarial antigen at six months of age compared with infants who did not develop severe malaria.

## Author summary

In this paper, we used a multiplexed, serologic assessment to identify children with previous or current exposure to or infection with filarial parasites or *S*. *stercoralis* (a soil transmitted helminth), enhancing our understanding of co-infections in early childhood. We identified an increasing prevalence of filarial antibodies over time in a population of children as young as 6 months old. In addition, we found a significant association between filarial seropositivity at six months of age and subsequent severe malaria.

## Introduction

Parasitic helminths and malaria are both highly prevalent globally and overlap extensively in tropical areas [1–3]. In 2016, more than 216 million cases of malaria were estimated to occur with 89 percent of cases occurring in Sub-Saharan Africa [4]. Nearly all cases of severe malaria are due to infection from *Plasmodium falciparum* [5], although *P*. *vivax* is increasingly regarded as a potential cause of severe malaria infection [6].

Lymphatic filariasis, caused exclusively by the helminth *Wuchereria bancrofti* in Africa, affects an estimated 120 million to 130 million persons globally [7]. *W*. *bancrofti* is highly endemic throughout Tanzania and especially in the northeast region [8–10], with an estimated 34 million people at risk of filarial infection and 6 million people affected by filariasis [11]. Filariasis has an overlapping geographical distribution with malaria in Tanzania [12–14] where it shares the same Anopheles vector as *P*. *falciparum* [15]. Co-infection is frequent [16, 17], with between 0–11% of school age children co-infected, depending on local ecology, in one study from Mvomero District, Tanzania [16]. In 2000 the Tanzanian National Lymphatic Filariasis Elimination Programme (NLFEP) was launched to distribute ivermectin and albendazole, highlighting the disease burden in this region [18], however the program is limited to individuals greater than 5 years of age.

The interaction of helminth and malaria co-infections is not well understood, and studies have had contradictory conclusions related to the inter-infection effects [19–23]. Differences in *W*. *bancrofti* prevalence by age have been previously described [16, 24, 25], but few studies have focused on filarial infection among infants, who are most likely to suffer severe malaria in the context of high malaria endemicity. The effects of *W*. *bancrofti* infection on the risk of malaria [12], especially severe malaria among infants, are largely unknown despite the findings of filarial-induced immune modulation on malaria-specific responses [26–28].

To address this gap, we used the remaining serum samples from infants and children in the Mother-Offspring Malaria Study (MOMS) Project, a large cohort study conducted from 2002 to 2006 in Muheza, Tanzania, to estimate the effect of exposure to filarial parasites on subsequent severe malaria infections. We tested the sera for reactivity to crude filarial antigens using a well-established immunoassay. We hypothesized that coinfection with filarial and *Plasmodium* species will modify immune responses and impact the risk of severe malaria.

## Materials and methods

Details for the cohort have been previously described [29]. Briefly, starting at birth, serum samples were taken at 3 and 6 months (+/- 2 weeks) of age and then at 6-month intervals, and malaria smears were collected at 2-week intervals during infancy and at 4-week intervals thereafter. All data analyzed were de-identified and anonymized. We used the remaining samples from this study to perform the assays listed below, along with the participant data already collected to perform this exploratory study. A comparison of participant characteristics between the previously published study and the current study is provided in Table 1.

**Table 1:** Characteristics of children and samples included in the study.

Characteristic	Goncalves et al. 2014 N = 882	This Study N = 429
**Newborn Sex**		
Male	457 (51.8)	215 (50.1)
Female	425 (48.2)	214 (49.9)
**Maternal Parity**		
Primi	254 (28.8)	116 (27.0)
Secundi	201 (22.8)	94 (21.9)
Multi	427 (48.4)	219 (51.1)
**Placental Malaria**		
PM+	116 (13.2)	52 (12.1)
PM-	766 (86.8)	377 (87.9)
**Transmission season at birth**		
High	432 (49.0)	194 (45.2)
Low	450 (51.0)	235 (54.8)

**Fig 1:**
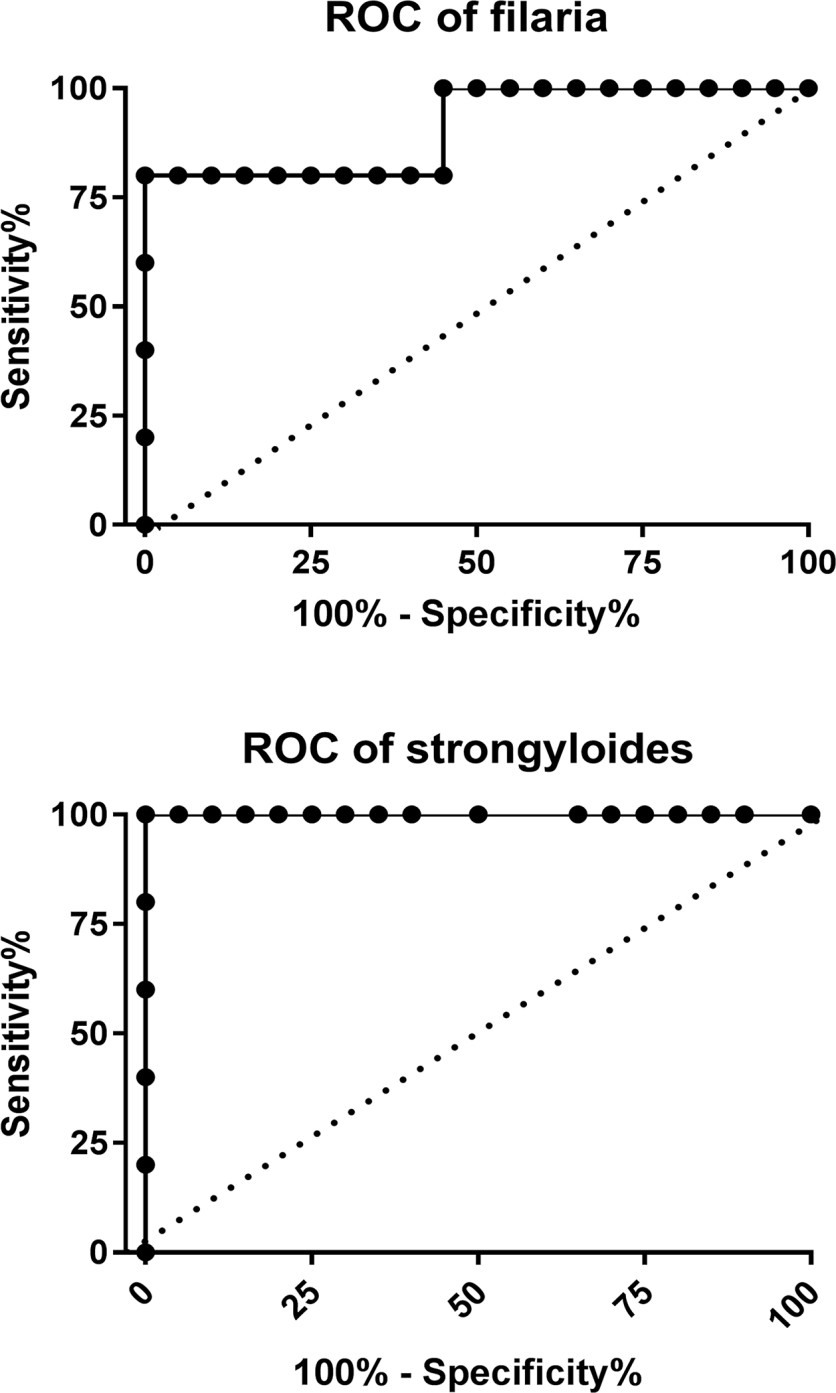
**Receiver operating curves for filaria (a), Strongyloides (b) using sera from US naïve donors as negative controls and from parasitologically proven infected donors as positive controls.** The analysis suggested good performance characteristics for both assays, with optimal cutoff values for seropositivity of 107 for filaria and 170 for Strongyloides.

Filarial-specific antibody levels were measured using a multiplex array system modified for filarial and Strongyloides antigen from a technique published by Fouda *et al* [30] for *P*. *falciparum*. Briefly, crude soluble lysates from *Brugia malayi* adults (BmA) or *S*. *stercoralis* larvae were coupled to fluorescently labeled beads. *B*. *malayi* antigen is used for filarial assays owing to its high cross-reactive antigenicity with *W*. *bancrofti* [31], and its amenability to in vitro culture to generate assay antigen. Ten positive control sera were collected from parasitologically proven infections with *W*. *bancrofti* (for BmA) (n = 5) and *S*. *stercoralis* (for Strongyloides) (n = 5). Negative control sera were from 19 non-exposed adults in the United States. Samples were assayed in duplicate. Discrepancies between duplicates were evaluated by the coefficient of variance: the highest result was dropped from pairs with a coefficient of variance greater than 0.4. The mean value of the duplicates was used where the coefficient of variance was less than 0.4. The positive cutoff was defined using a receiver operating characteristic (ROC) curve to identify the cutoff point that produced the highest sensitivity and specificity based on positive and negative controls (Fig 1).

Available serum samples from children ≤2.5 years of age were assayed for antibodies to filarial antigen and to *S*. *stercoralis* larval antigen. Subsequent risk factor analysis was further limited to visits at 6 months and 1 year of age because this was the age window during which most severe malaria infections occurred. Children were excluded from this analysis if they: 1) had HIV or sickle cell anemia; 2) were twins or triplets; 3) had moved away from the study area since enrollment. Severe malaria was defined using WHO criteria [32].

### Risk factor analysis

Logistic regression models were used to assess the risk of severe malaria in the 6 months following sample collection for measurement of filarial serologies. We estimated the risk of severe malaria in the 6-month period following serologies to determine if children who were seropositive were at higher risk of severe malaria than those who were seronegative. To assess confounding, we estimated the association of specific variables known to be related to severe malaria (maternal parity, placental malaria, village, infant anemia, presence of an insecticide treated bed net in the household and malaria transmission season during birth) for their association with both filarial sero-status and severe malaria in this cohort. Only variables that were significantly associated with both severe malaria and filarial sero-status in this cohort were considered confounders. All models included only children who had a positive malaria blood smear in the six months following the visit when filarial serology was assessed, because development of severe malaria requires infection from *P*. *falciparum*. Statistical significance was assessed at p < 0.05.

### Cytokines

We quantified plasma cytokine levels at 6 months and 1 year of age to assess associations with filarial serology and severe malaria risk, after stratifying by malaria blood smear positivity. We assessed pro-inflammatory (IL1, IL6, IFNγ and TNFα) and anti-inflammatory cytokines (IL4, IL5, and IL10), based on the hypothesis that the balance of pro- and anti-inflammatory cytokine levels may influence severe malaria risk [33]. Cytokine assays were performed as previously described [34, 35]. The detection limits for the different analytes were as follows: TNF-α, 0.10 pg/ml; IFN-γ, 0.04 pg/ml; IL-1β, 0.01 pg/ml; IL-4, 0.3 pg/ml; IL-5, 0.02 pg/ml; IL-6, 1.45 pg/ml; IL-10, 0.02 pg/ml. Values were log transformed after adding one to all values to avoid log transformation of zero and the geometric means were compared using ANOVA. IL4 was analyzed as detectable vs. non-detectable using the detectable limit of 0.3. We accounted for multiple comparisons by using a Bonferroni correction. Statistical significance was assessed at p < 0.0125.

### Ethics

Data for the MOMS study were collected under protocols approved by the International Clinical Studies Review Committee of the Division of Microbiology and Infectious Diseases at the US National Institutes of Health, and ethical clearance was obtained from the Institutional Review Boards of Seattle BioMed and the National Medical Research Coordinating Committee in Tanzania.

## Results

**Fig 2:**
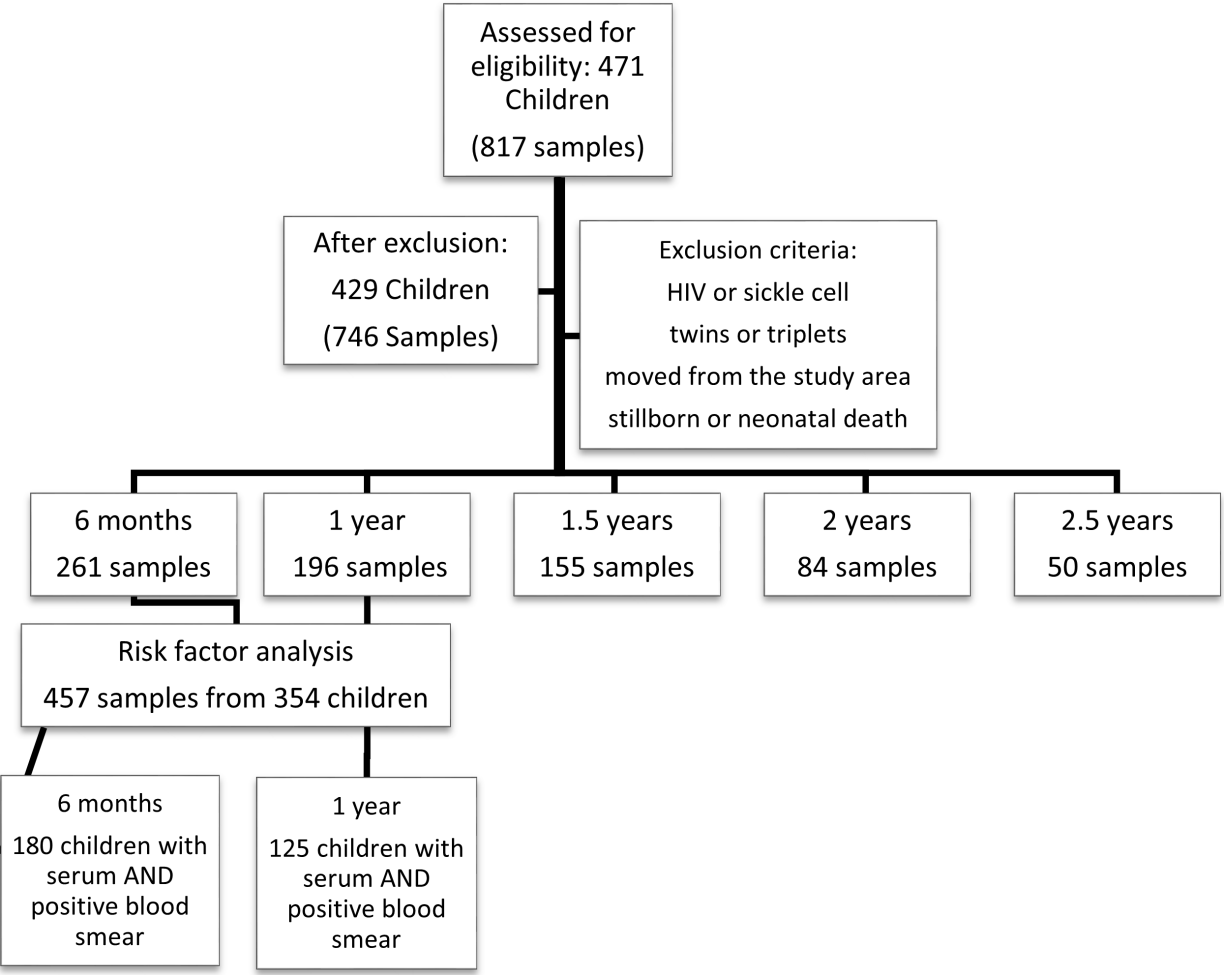
Flowchart of children and samples included in the study. A total of 471 children were assessed for eligibility in the study. Children were excluded from the study due to HIV or sickle cell anemia, if it was a multiple birth, or if they moved from the study area. Children with blood samples at 6 months (n = 261 samples) and 1 year (n = 196 samples) were used for the risk factor analysis, of which 180 children had serum for the assays and a positive blood smear between 6 months and one year of age, and 125 children had serum and a positive blood smear between one year and 18 months of age.

**Fig 3:**
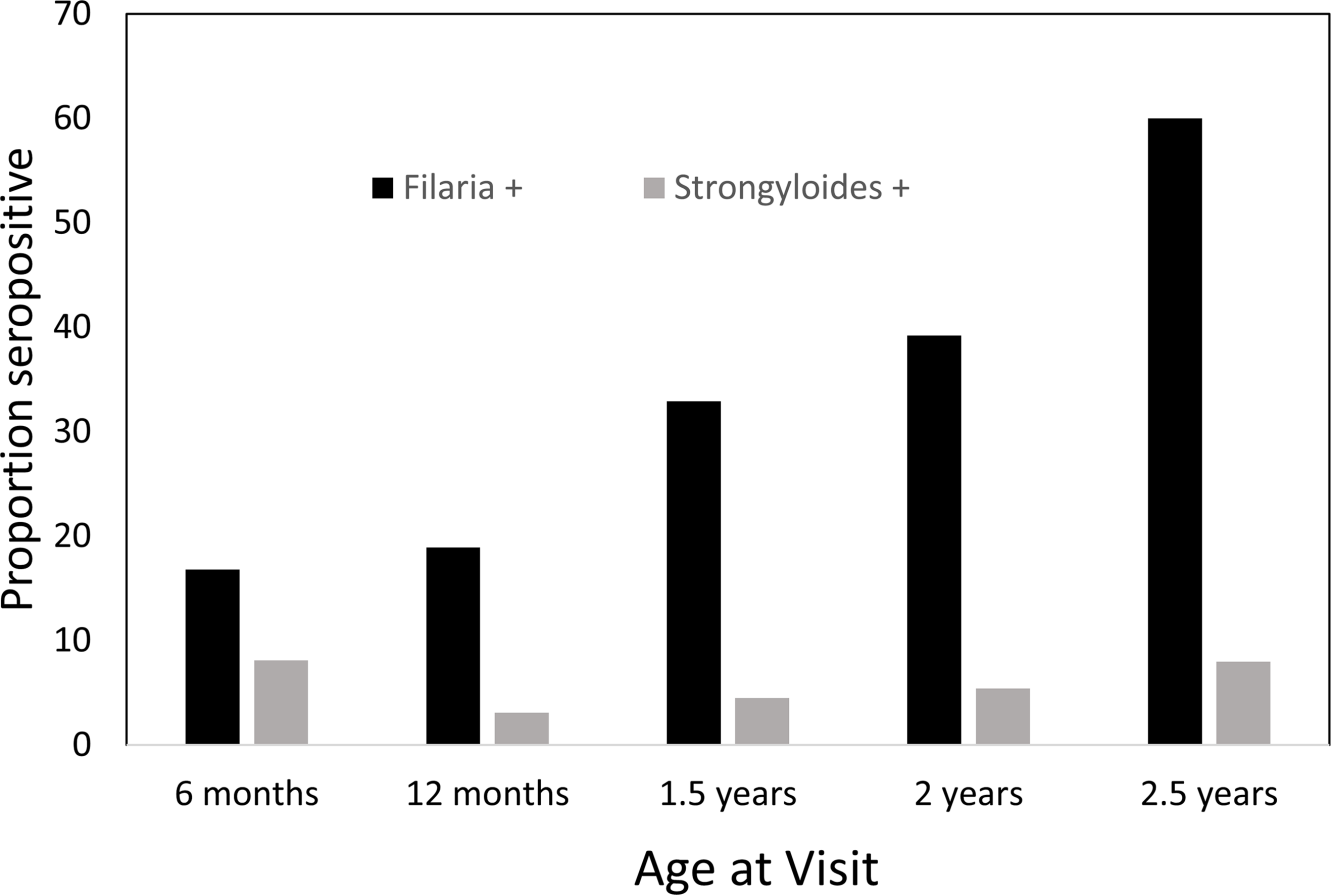
Proportions of children seropositive for filaria and for Strongyloides by age. The proportion of children with filarial antibodies increased with age: 16.8% at 6 months, 18.9% at one year, 32.9% at 1.5 years, 39.2% at 2 years to 60.0% at 2.5 years. In contrast, the proportion of children with antibodies to Strongyloides stayed fairly constant: 8.1% at 6 months, 3.1% at 1 year, 4.5% at 1.5 years, 5.4% at 2 years and 8.0% at 2.5 years.

A total of 746 serum samples were selected for risk factor analysis as outlined by the flowchart in Fig 2. The proportion of children with positive serology to filarial antigens ranged from 16.8%-60% with the highest proportion seropositive at 2.5 years. The percentage of children with positive serology to Strongyloides antigens ranged from 3.1–8.1% (Fig 3). Because of the low numbers of children with positive serology for Strongyloides, we did not include these in subsequent risk factor analysis (Table 2).

**Table 2:** Seroprevalence and blood smear status of children in the study, n (%).

	Serology and Blood Smear Status by Age n (%)				
	6 months	12 months	1.5 years	2 years	2.5 years
Filaria +	44 (16.8)	37 (18.9)	51 (32.9)	40 (39.2)	30 (60.0)
Strongyloides +	21 (8.1)	6 (3.1)	7 (4.5)	6 (5.4)	4 (8.0)
Blood smear positive (%)	27 (10.3)	23 (11.8)	16 (10.7)	14 (16.7)	4 (8.7)
Average Parasites (SD)*	244.6 (767.8)	318.5 (559.4)	1492.0 (4002.9)	172.0 (301.8)	480.7 (688.2)

*Positive samples only. Parasite count is per 200 WBCs.

**Fig 4:**
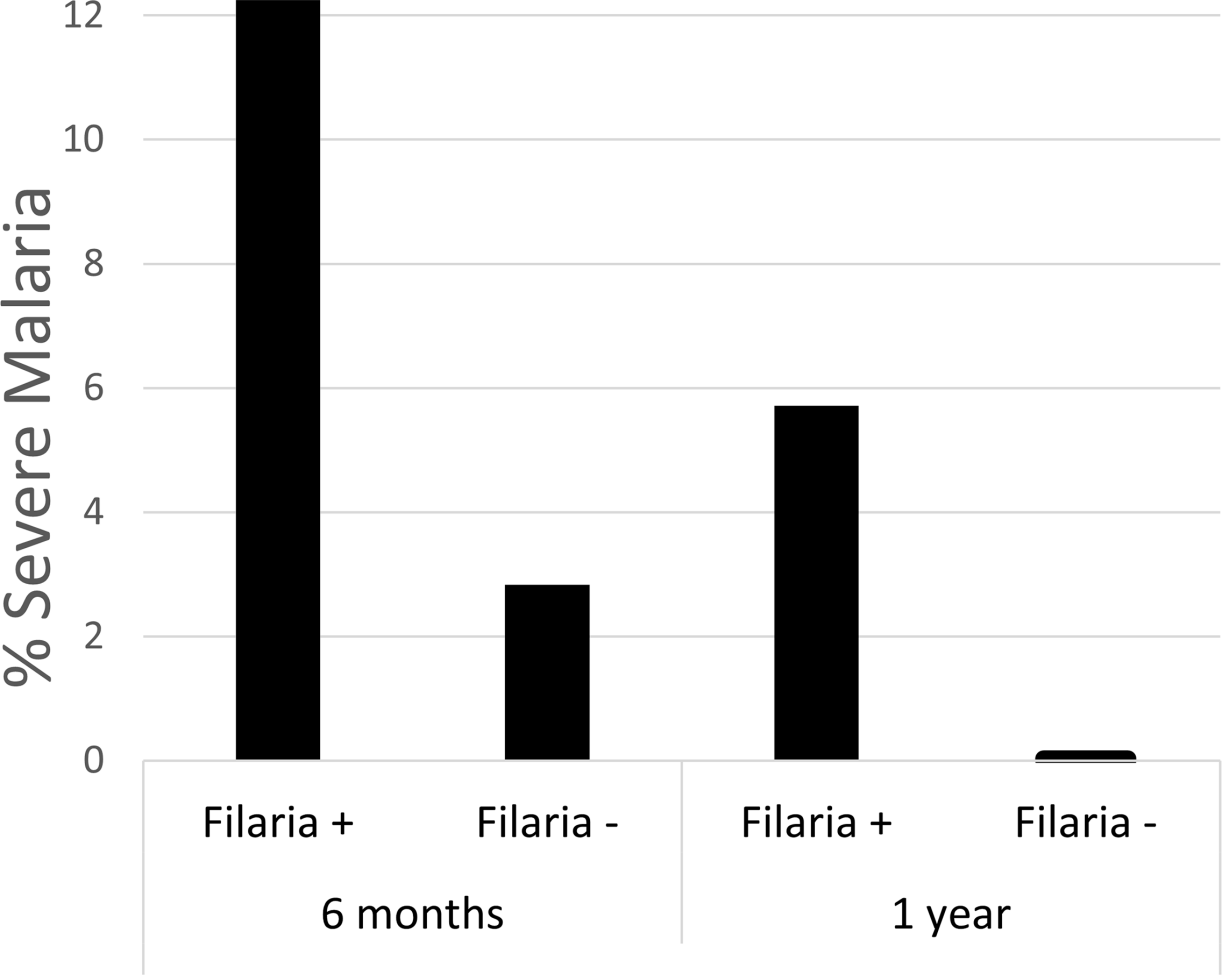
Proportions of children who subsequently developed severe malaria by filaria serostatus and age. The proportion of children with filarial antibodies who developed severe malaria over the subsequent 6 months was 12.2% at 6 months of age and 5.7% at one year. The proportions of children without filarial antibodies who developed severe malaria over the ensuing 6 months was lower: 2.83% at 6 months and 0.04% at one year. No subsequent severe malaria events were observed at time points past 18 months of age in these subjects.

Children with remaining serum samples collected between 6 months and 1.5 years of age (n = 612) were assessed for the occurrence of severe malaria and serologic evidence of filarial infection, with the highest proportion of severe malaria events occurring in the first year of life (Fig 4).

In order to assess risk associated with filarial serology, we used the data for children with samples at 6 months and 1 year who also had blood smear data. To better understand the risk of progressing to severe malaria among those already infected, we further limited the sample to children with at least one positive blood smear: 236 children had at least one serum sample at 6 months or 1 year of age and subsequent or concurrent positive malaria blood smear. 180 children had a serum sample at 6 months of age and a subsequent or concurrent positive malaria blood smear, and 125 children had a serum sample at 1 year of age and a subsequent positive malaria blood smear. Overall, of the 236 children, 70% (n = 166) had a report of treated bed net use at some period during the observation period and 16% (n = 38) had missing information for this variable.

### Risk factor analysis

None of the tested potential confounding variables were associated with both severe malaria and filarial antibody positivity in this cohort. We assessed the risk of severe malaria in the 6 months after filarial seropositivity at 6 months and 1 year. We found that infants who developed severe malaria between 6 months and 1 year of age were 3.9 times more likely (OR = 3.9, 95% CI: 1.2, 13.0; p-value = 0.02) to have had positive filarial serology at 6 months of age compared with infants who did not develop severe malaria. Children with severe malaria between 1 and 1.5 years of age were not significantly more likely to have positive filarial serology at 1 year of age than children who did not develop severe malaria (OR = 1.4; 95% CI: 0.27, 7.6; p-value = 0.67).

### Cytokine analysis

**Fig 5:**
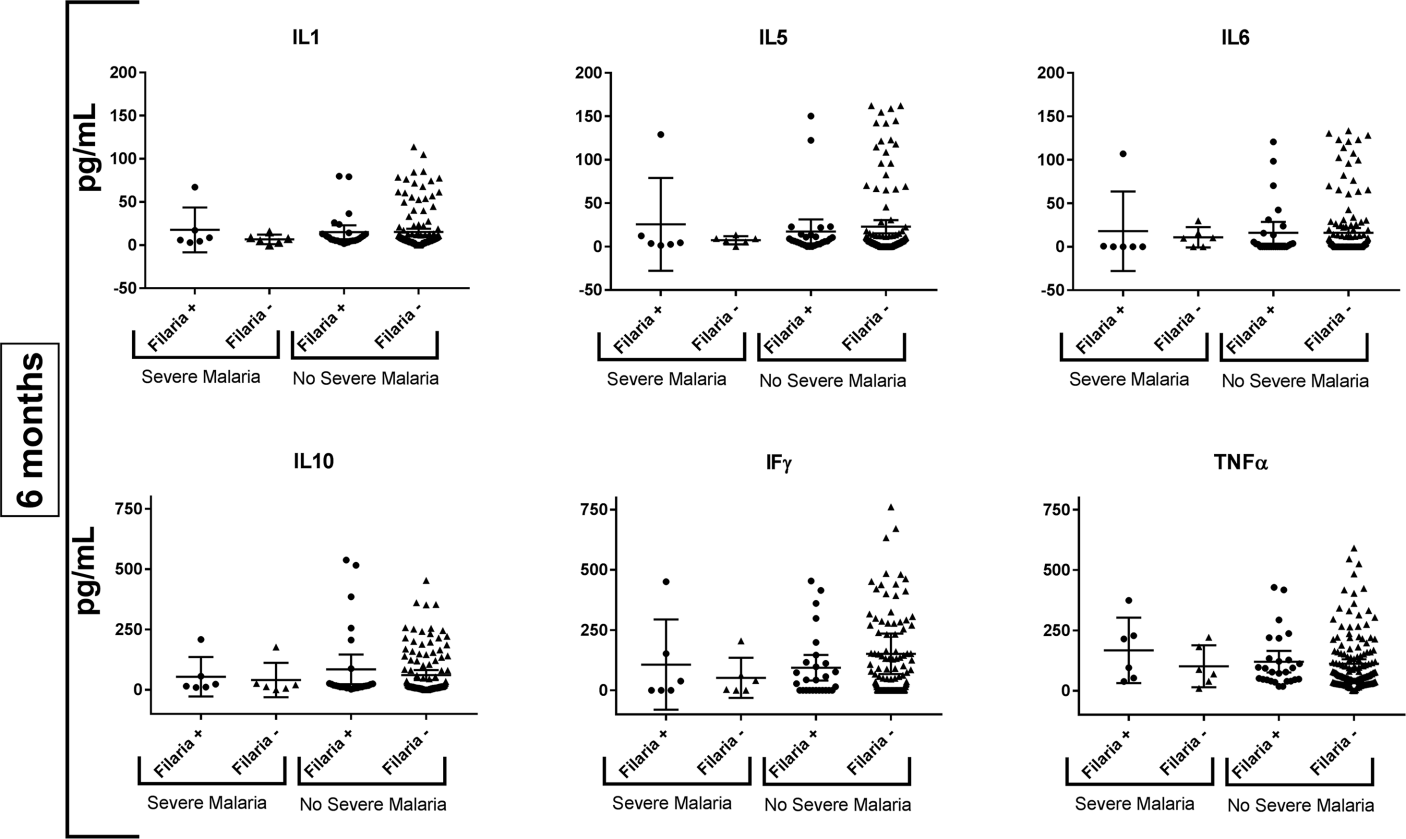
Cytokine profiles of children in the study at 6 months by filarial serostatus, stratified by whether they experienced (“Severe Malaria”) or did not experience (“No Severe Malaria”) severe malaria between 6 and 12 months of age. No significant differences in cytokine levels were detected for children who did versus did not develop severe malaria.

**Fig 6:**
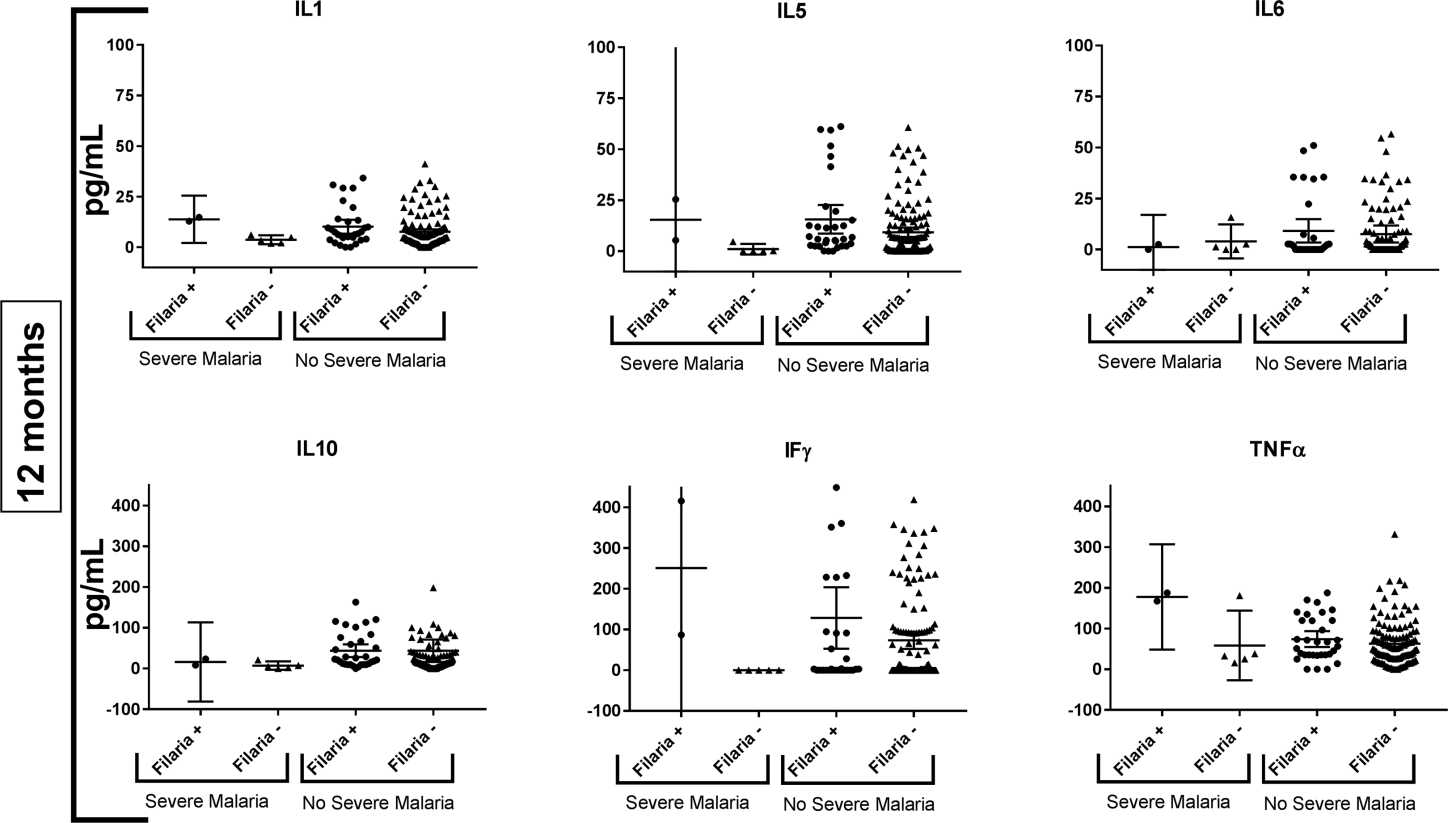
Cytokine profiles of children in the study at 12 months by filarial serostatus, stratified by whether they experienced (“Severe Malaria”) or did not experience (“No Severe Malaria”) severe malaria between 12 and 18 months of age.

We did not identify a difference between IL1, IL5, IL6, IL10, TNF-alpha, and IFN-gamma levels by filarial serology at 6 months or 1 year of age when using a significance value of p< 0.0125 among infants who were blood smear positive or negative. Among infants who subsequently developed severe malaria, no significant difference in IL1, IL5, IL6, IL10, TNF-alpha, or IFN-gamma levels by filarial serology was found at either 6 months or 1 year of age (Figs 5 and 6). Likewise, the presence of detectable IL4 was not associated with severe malaria risk during the 6 months after assay (Table 3).

**Table 3:** Presence of detectable IL4 by severe malaria diagnosis and filarial serology status, n(%).

	6 months				1 year			
	Severe Malaria		No Severe Malaria		Severe Malaria		No Severe Malaria	
	Filaria+	Filaria-	Filaria+	Filaria-	Filaria+	Filaria-	Filaria+	Filaria-
IL4 detectable	1 (16.7)	1 (16.7)	5 (18.5)	36 (24.2)	2 (100)	0 (0)	11 (34.4)	25 (17.9)
IL4 not detected	5 (83.3)	5 (83.3)	22 (81.5)	113 (75.8)	0 (0)	5 (100)	21 (65.6)	115 (82.1)

## Discussion

We describe an age-specific increase in prevalence of filarial antibodies beginning in infancy in Tanzania. Although previous studies have identified filarial infection in young children, this study identifies an increasing filarial seropositivity with age starting at 1 year. Additionally, we found that filarial seropositivity at 6 months of age was significantly associated with severe malaria by 1 year of age.

Although transplacental maternal antibodies may play a role in the observed prevalence, particularly at the 6-month measurement, the increasing seroprevalence after 1 year is noteworthy. Weerasooriya *et al*. described a decline in urinary antigens after 1 year of age among infants born to mothers who were *Brugia pahangi* antibody-positive [36]; antibodies from breast milk are not known to enter the infant’s circulation [37] suggesting that our results in children 1 year and older indicate the presence of antibodies acquired through filarial exposure rather than through maternal-infant transfer. Many public health studies focus on school age children when describing infections among children. However, our results may indicate that pre-school age children are at increased risk of filarial exposure as well.

This study is also valuable in that it uses a multiplexed serologic assessment to identify children with previous or current exposure or infection for filariae and Strongyloides. The assay data appear consistent using the non-exposed controls, so we are confident that we are detecting both exposures. This obviously provides a framework for adding multiple (up to 50) antigens to gain a comprehensive assessment of seroreactivity in a single assay [38]. Other helminths contribute to polyparasitism in Tanzania including: *Onchocerca volvulus*, the causative agent of onchocerciasis [39]; *S*. *mansoni* and *S*. *haematobium* [40] [41]; and multiple species of soil-transmitted helminths. Several challenges exist with identifying the soil-transmitted helminth *S*. *stercoralis* infections in young children, a focus of this study. Very young children are incorrectly thought to be at lower risk based on the idea that they are not in direct contact with infective sources. Further, diagnosis may be hindered because measures of active infection (e.g. eggs in stool, parasites in the blood) may lag behind serologic measures of exposure [42].

The significant association between filarial serostatus at 6 months and subsequent severe malaria infection highlights the need for further investigation to assess whether the increased risk is due to a shared vector or if immune modulation is occurring. As such, a primary limitation to this analysis is the potential for confounders in the relationship between severe malaria and filarial serology. Both malaria and filarial parasites are transmitted by mosquitoes, and previous studies have suggested that the same mosquito species may transmit both infections [17, 41].

The association between filarial seropositivity and severe malaria has been reported elsewhere in studies of older children and adults. Increased risk of severe malaria with helminth co-infection has been reported in children aged between 1–15 years in Senegal and Northern Senegal, and helminth coinfection has been associated with an increase in clinical malaria in children aged < 16 years in Zaire [23, 43, 44]. A study of adults in Thailand found an increase in clinical malaria associated with co-infection with intestinal helminths [19]. Conversely, studies in Senegal and Mali found decreases in malaria parasite densities associated with *S*. *haematobium* co-infection in cohorts aged 3–15 [21, 45], and no influence on malaria incidence was found in mixed age cohorts in Southwest Uganda and Northern Senegal [46, 47]. These apparent differences in findings may be explained by parasite differences, with *S*. *haematobium* co-infections having little or no influence on severe malaria, while Ascaris, *S*. *mansoni*, filarial and Strongyloides co-infection may confer increased risk. The differences may also be attributable to the age of the children in this study, as the children in this cohort are younger and the effect of coinfection may be different, or the antibodies present may reflect maternal antibodies.

We assessed potential confounding for a variable indicating the presence of an insecticide treated bednet in the household and this variable was not significantly associated with filarial serology at 6 months or 1 year of age. A modest proportion of children were missing information for this variable. Even among children who had an insecticide treated net in their household, we are unaware of actual utilization rates or integrity of the bed nets, so actual assessment of treated bed net use may be imprecise.

This study has several additional limitations. First, we are unable to determine if the positive filarial serology indicates current or past infection. Because we do not have measures of current infection, we are unable to determine if our results correlate with infection or exposure and are limited to describing the associations with filarial sero-reactivity. However, one advantage of using serology is that we are able to observe cumulative exposure, rather than assessing exposure at a single point in time. Secondly, this well-established serologic assay specifically uses *Brugia malayi* antigens, based on the substantial antigenic cross-reactivity among all filarial species [8–10, 39], and as a result this assesses filarial exposure without assignment to an exact species. However, the age-specific profile aligns much more closely with *W*. *bancrofti* than with *O*. *volvulus* infection [48–51] as does the geospatial data. Although we now have filarial species-specific recombinants for *W*. *bancrofti* [52–54] and *O*. *volvulus* [55], insufficient serum was available to perform these specific assays. Nonetheless, the results still suggest filarial antibodies are an important biomarker of increased risk for severe malaria and further suggest that efforts to reduce exposure to the vectors associated with *W*. *bancrofti* or *O*. *volvulus* infection may also have a substantial impact on reducing severe malaria.

## Supporting information

S1 Table Samples per participant, by timepoint used in the analysis. Samples were the remaining sera from the Mother-Offspring Malaria Study (MOMS) Project conducted from 2002 to 2006 in Muheza, Tanzania.
(TIF)
