# Supplementary figures and images for: Seroepidemiology of helminths and the association with severe malaria among infants and young children in Tanzania
Source: PLoS Negl Trop Dis. 2018 Mar 26;12(3):e0006345. doi: 10.1371/journal.pntd.0006345 (PMC5886694; doi:10.1371/journal.pntd.0006345)

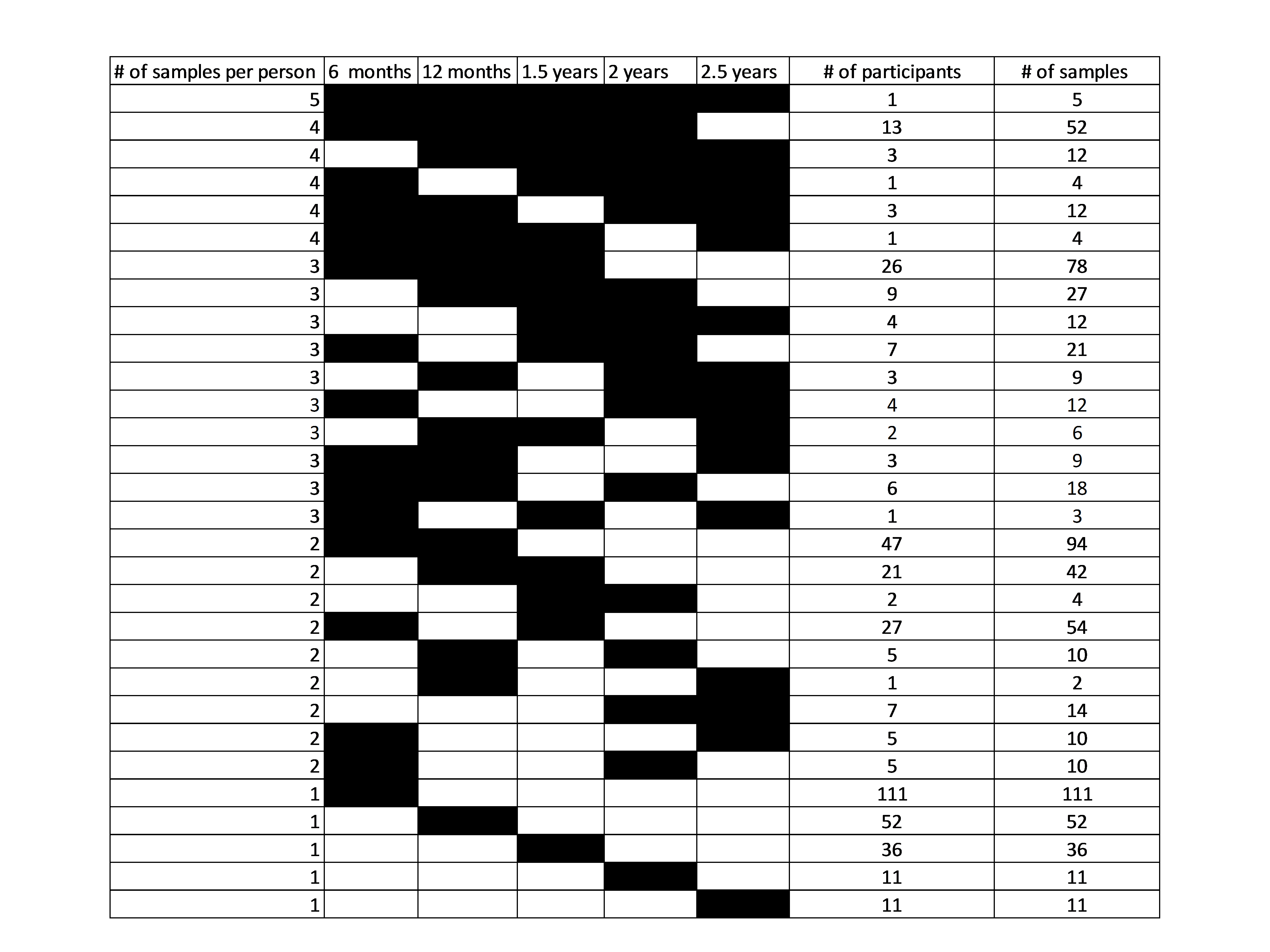

Supplement: S1 Table — Samples were the remaining sera from the Mother-Offspring Malaria Study (MOMS) Project conducted from 2002 to 2006 in Muheza, Tanzania. (TIF) [file pntd.0006345.s001.TIF]
